# The potential importance of unburned islands as refugia for the persistence of wildlife species in fire‐prone ecosystems

**DOI:** 10.1002/ece3.5432

**Published:** 2019-07-04

**Authors:** Jasper Steenvoorden, Arjan J. H. Meddens, Anthony J. Martinez, Lee J. Foster, W. Daniel Kissling

**Affiliations:** ^1^ Institute for Biodiversity and Ecosystem Dynamics (IBED) University of Amsterdam Amsterdam The Netherlands; ^2^ Department of Natural Resources and Society University of Idaho Moscow ID USA; ^3^ Oregon Department of Fish and Wildlife Hines OR USA

**Keywords:** conservation, disturbance, fire refugia, sagebrush ecosystem, unburned islands, wildfire

## Abstract

1. The persistence of wildlife species in fire‐prone ecosystems is under increasing pressure from global change, including alterations in fire regimes caused by climate change. However, unburned islands might act to mitigate negative effects of fire on wildlife populations by providing habitat in which species can survive and recolonize burned areas. Nevertheless, the characteristics of unburned islands and their role as potential refugia for the postfire population dynamics of wildlife species remain poorly understood.
2. We used a newly developed unburned island database of the northwestern United States from 1984 to 2014 to assess the postfire response of the greater sage‐grouse (*Centrocercus urophasianus*), a large gallinaceous bird inhabiting the sagebrush ecosystems of North America, in which wildfires are common. Specifically, we tested whether prefire and postfire male attendance trends at mating locations (leks) differed between burned and unburned areas, and to what extent postfire habitat composition at multiple scales could explain such trends.
3. Using time‐series of male counts at leks together with spatially explicit fire history information, we modeled whether male attendance was negatively affected by fire events. Results revealed that burned leks often exhibit sustained decline in male attendance, whereas leks within unburned islands or >1.5 km away from fire perimeters tend to show stable or increasing trends.
4. Analyses of postfire habitat composition further revealed that sagebrush vegetation height within 0.8 km around leks, as well elevation within 0.8 km, 6.4 km, and 18 km around leks, had a positive effect on male attendance trends. Moreover, the proportion of the landscape with cheatgrass (*Bromus tectorum*) cover >8% had negative effects on male attendance trends within 0.8 km, 6.4 km, and 18 km of leks, respectively.
5. *Synthesis and applications*. Our results indicate that maintaining areas of unburned vegetation within and outside fire perimeters may be crucial for sustaining sage‐grouse populations following wildfire. The role of unburned islands as fire refugia requires more attention in wildlife management and conservation planning because their creation, protection, and maintenance may positively affect wildlife population dynamics in fire‐prone ecosystems.

## 1 INTRODUCTION

Fire has structured the distribution of ecosystems and their wildlife species for millions of years (Bond & Keeley, 2005). Nevertheless, the persistence of species in fire‐prone ecosystems is under increasing pressure from global environmental change, including alterations in fire regimes caused by climate change and anthropogenic activity (Flannigan, Krawchuk, De Groot, Wotton, & Gowman, 2009). Under changing fire regimes, present‐day fire impacts on wildlife species are potentially greater than those experienced by species during their evolutionary history (Brook, Sodhi, & Bradshaw, 2008). However, as fire is an inherently heterogeneous disturbance, specific components of the landscape may endure after fire (Burton, Parisien, Hicke, Hall, & Freeburn, 2008). Such unburned islands of vegetation could serve as refugia (Meddens, Kolden, Lutz, Abatzoglou, & Hudak, 2018; Meddens, Kolden, Lutz, Smith, et al., 2018) and may mitigate negative impacts of fire disturbances on wildlife species by providing habitat in which species can survive and from which they can recolonize burned areas, thus increasing their likelihood of persistence (Keppel et al., 2012; Robinson et al., 2013). Yet, the importance of unburned islands for conservation and management of wildlife populations remains poorly understood (Kolden, Lutz, Key, Kane, & van Wagtendonk, 2012).

In the Great Basin of North America, wildfires are a common component of terrestrial ecosystems. The natural vegetation is often dominated by sagebrush (*Artemisia tridentata*), a shrub species adapted to arid and semi‐arid conditions. Sagebrush has long recovery times (35–120 years) after fire (Baker, 2006). As a result, the invasion of exotic grasses like cheatgrass (*Bromus tectorum*) and trees like western juniper (*Juniperis occidentalis*, hereafter juniper) in combination with climate change, fire suppression, and urbanization (Connelly, Knick, Schroeder, & Stiver, 2004; Murphy et al., 2013) has strongly changed fire regimes in sagebrush ecosystems. These phenomena have caused changes in the frequency, size, and intensity of fires, which may now rapidly reach catastrophic sizes with detrimental effects on ecosystem functioning (Miller et al., 2011). Identifying and understanding the spatial distribution, habitat characteristics, and ecological role of unburned islands for preserving wildlife species and ecological functions of ecosystems is therefore becoming increasingly important for habitat and natural resource management (Meddens, Kolden, & Lutz, 2016).

The greater sage‐grouse (*Centrocercus urophasianus*: hereafter, sage‐grouse) is a sagebrush‐obligate bird species widely distributed throughout the Great Basin of North America. However, the species has experienced persistent population declines over the last half century, largely as a result of habitat loss and degradation (Garton et al., 2011; Schroeder et al., 2004). Although the specific effects of wildfire on sage‐grouse population dynamics and habitat selection remain largely unknown (Foster, 2016), life‐history traits and ecological requirements of sage‐grouse may give some indication about how populations of this species respond to fire disturbances (Connelly, Rinkes, & Braun, 2011; Crawford et al., 2004). Sage‐grouse exhibits strong site fidelity and individuals typically return to the same lek (mating location) or groups of leks on a yearly basis (Fremgen et al., 2017). Due to this high philopatry, sage‐grouse may remain in fire disturbed habitat and attempt to select habitat at the microscale to meet their life‐history requirements, even if this behavior has severe fitness costs (Foster, 2016). Second, sage‐grouse require widespread and intact sagebrush habitats for food and shelter from predators during all stages of their life (Crawford et al., 2004; Hagen, 2011b). Therefore, the extent and characteristics of sagebrush habitat are directly linked to the persistence of this species (Knick, Hanser, & Preston, 2013). Observed declines in sage‐grouse populations following wildfire are most likely caused by reduced survival and reproduction through loss of habitat, rather than due to emigration away from fire‐affected areas (Coates et al., 2015; Foster, Dugger, Hagen, & Budeau, 2019).

**Figure 1:**
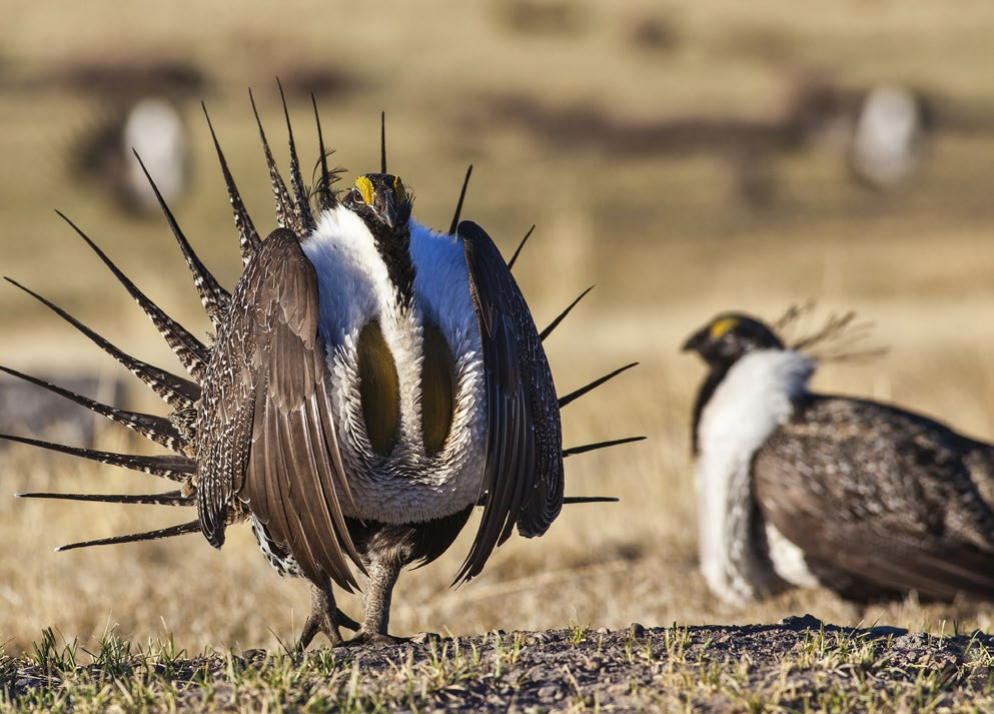
Male sage‐grouse (*Centrocercus urophasianus*) strutting on a lek. Image courtesy of Bureau of Land Management (available with a CC BY license)

**Figure 2:**
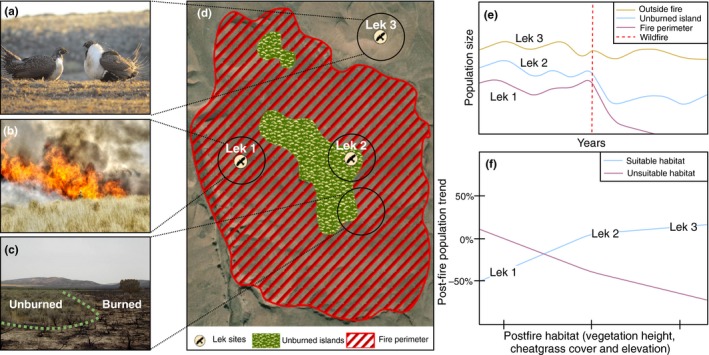
Fire dynamics in the sagebrush ecosystem and hypothesized postfire responses of the sage‐grouse to burned and unburned areas. (a) The greater sage‐grouse (*Centrocercus urophasianus*), a characteristic wildlife species of the sagebrush ecosystem in the Great Basin of North America. (b) Wildfires are common in this ecosystem and result (c) in a mosaic of burned and unburned islands. (d) At the landscape scale, lek sites (i.e., mating locations) of the sage‐grouse might be located within fire perimeters (lek 1), in unburned islands inside fire perimeters (lek 2), or outside fire perimeters (lek 3). (e) Depending on the location of leks in burned or unburned areas, postfire population trends (growth/decline per year) might be negative (inside fire perimeters) or relatively stable (within unburned islands or outside fire perimeters). (f) The amount of postfire habitat surrounding lek sites might affect postfire population trends positively (availability of suitable sagebrush vegetation) or negatively (amount of unsuitable cheatgrass cover). Images (a) courtesy of Sarah McIntire, University of Idaho, (b) courtesy of Bureau of Land Management (available with a CC BY license), (c) adapted from Jones, Monaco, and Rigby (2015)

Here, we focus on the potential role of unburned islands for the persistence of the sage‐grouse (Figure 1) within sagebrush ecosystems of the Great Basin. Our aim was to analyze temporal (≥6 years) trends in male lek attendance of sage‐grouse (Figure 2a) in relation to wildfires (Figure 2b) and burned and unburned areas (Figure 2c). Specifically, we tested whether prefire and postfire male attendance trends at leks differ between burned and unburned areas (Figure 2d), and to what extent postfire habitat characteristics could explain such trends (Figure 2e,f). Based on current knowledge about fire refugia and the ecological requirements of sage‐grouse, we test two hypotheses: (a) unburned islands within fire perimeters as well as unburned areas outside fire perimeters mitigate the negative effects of fire on male lek attendance of sage‐grouse by allowing stable or positive population trends (Figure 2e), and (b) postfire habitat composition surrounding leks will influence population trends after fire, either positively (via availability of suitable habitat) or negatively (via unsuitable habitat; Figure 2f). Assessing the importance of unburned islands as potential fire refugia for the persistence of wildlife species is of key importance, as there are still major knowledge gaps in understanding the spatiotemporal dynamics of wildlife populations within postfire vegetation mosaics (Robinson et al., 2013).

## 2 MATERIALS AND METHODS

### 2.1 Study area

**Figure 3:**
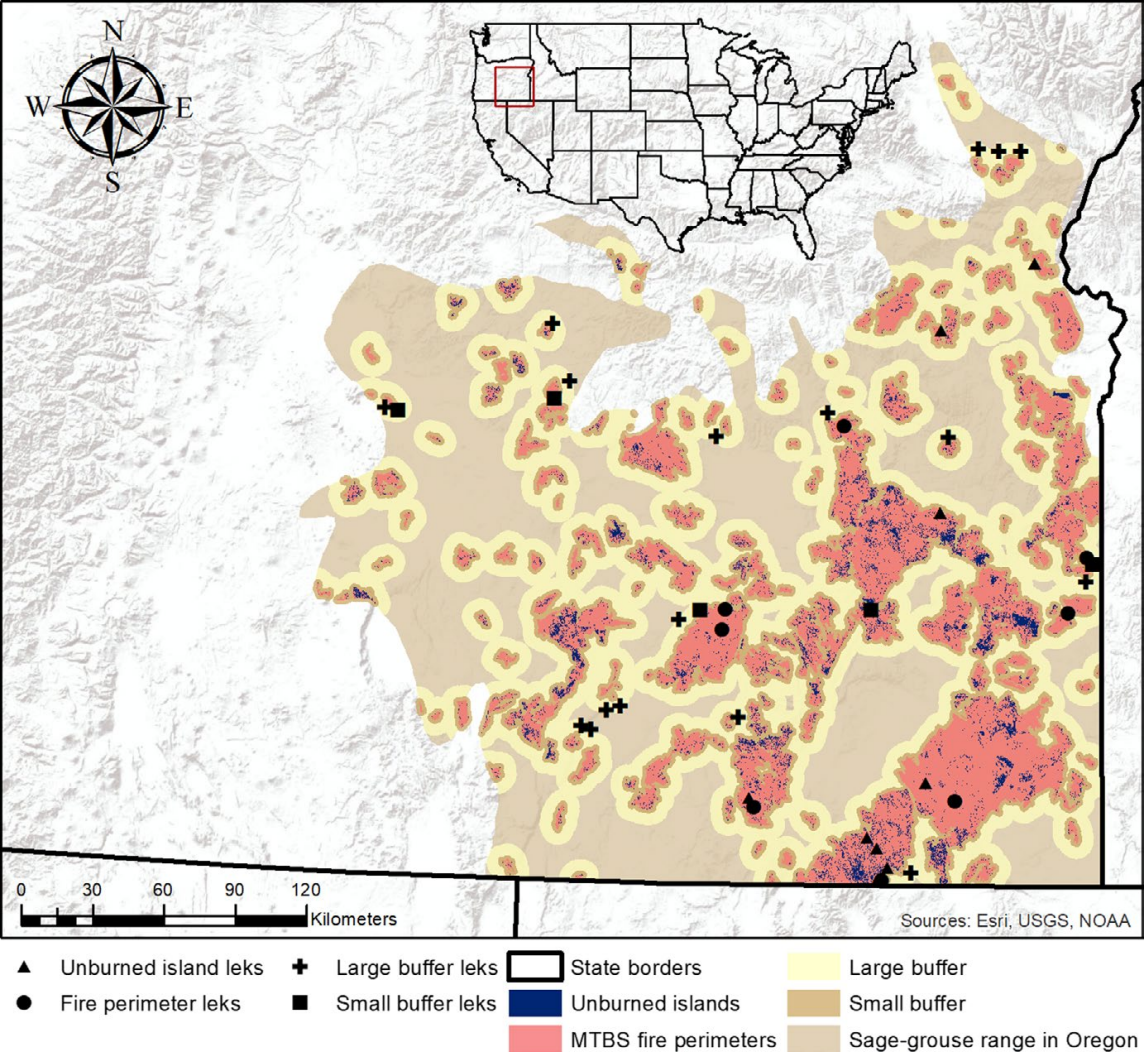
Study area showing the fire history within the geographic range of the greater sage‐grouse (*Centrocercus urophasianus*) in southeastern Oregon, USA (retrieved from the ODFW, 2017). The effect of fire on the landscape is represented with unburned islands, fire perimeters, small buffers (50 m–1.5 km from fire perimeters), and large buffers (1.5–6.4 km from fire perimeters). Analyzed leks (i.e., mating locations) of the sage‐grouse are shown within these four fire categories. The fire categories are based on a fire perimeter dataset from the Monitoring Trends in Burn Severity (MTBS) program (Eidenshink et al., 2007) and a derived unburned island database (Meddens, Kolden, Lutz, Abatzoglou, et al., 2018). The inset shows the extent of the study area (indicated as a red square) within the USA

The extent of the study area was defined by the geographical range of the sage‐grouse in Oregon, USA (Figure 3, ODFW, *unpublished data*, adapted from Schroeder et al., 2004). This represents an area of 47,257 km^2^ and is mostly dominated by sagebrush communities, grasslands, patches of conifer forest, and some agricultural areas. Fires are frequent in the study area, with 317 documented fires >405 ha from 1984 to 2014 (MTBS, 2014). The study area ranges in elevation between 1,120 and 2,750 m. Annual temperature ranges from −6 to 13°C, and annual precipitation averages between 200 and 700 mm (WorldClim, 2017).

### 2.2 Fire perimeters and unburned islands

We obtained fire perimeters for the study area between 1984 and 2014 from the MTBS program. This program records data on the date, size, extent, and severity class of each fire >405 ha documented in the western United States since 1984 (Eidenshink et al., 2007). Using the MTBS fire perimeter dataset, Meddens, Kolden, Lutz, Abatzoglou, et al. (2018) developed an unburned island database (30 m resolution) that contains the size and extent of unburned islands within fire perimeters in the states of Idaho, Washington, and Oregon, east of the Cascade crest. The fire perimeter and unburned island datasets were used to categorize leks in the study area for the population trend analysis, as well as to document the year in which leks or areas around leks were burned. We distinguished four fire categories: (a) *unburned island*, for leks inside unburned islands that are located within the fire perimeter, or leks close (<50 m) to an unburned island or fire perimeter; (b) *fire perimeter*, for leks located inside burned areas of the fire perimeter; (c) *small buffer* (50 m–1.5 km away from fire perimeters); and (d) *large buffer* (>1.5–6.4 km away from fire perimeters). We differentiated between small and large buffers outside fire perimeters to test if lek populations in the surroundings of wildfires were also affected. Leks in the small buffer area may show similar trends to unburned islands, whereas the large buffer category should represent lek populations that are unaffected by fire (control). The outer radius of the large buffer (6.4 km) was chosen because >80% of female sage‐grouse distribute their nests in an area of 6.4 km around leks (Doherty, Naugle, & Walker, 2010; Hagen, 2011a). Consequently, population dynamics of leks in this category could still be influenced by fire dynamics, but we expect those effects on population trends to be negligible.

### 2.3 Sage‐grouse lek count data

We used yearly peak male sage‐grouse counts of leks in the study area between 1984 and 2017 to analyze trends in male lek attendance. Data were obtained from the Oregon Department of Fish and Wildlife (ODFW) which monitors sage‐grouse populations in Oregon. Leks typically occur in the same locations each year (Connelly, Hagen, & Schroeder, 2011), and male sage‐grouse rarely move away between leks during the breeding season (Fremgen et al., 2017). When leks were situated within a lek complex (a group of multiple leks situated in close proximity), leks were counted on the same day to minimize potential movement of males between leks within the same lek complex. Currently, lek counts are the only data available for examining large‐scale trends in sage‐grouse population size and their spatial distribution (Connelly et al., 2004). However, many leks are not surveyed on a yearly basis and frequent surveillance of leks associated with specific fires often starts only after a fire. ODFW has used a standardized procedure to monitor sage‐grouse leks since 1996 (Hagen, 2011b). The resulting dataset of male lek attendance provides an index of population size from which temporal trends (i.e., growth or decline in male attendance) can be derived.

### 2.4 Population trend analysis

Leks were only included in our analysis if they: (a) were situated within one of the four fire categories (*unburned island*, *fire perimeter*, *small buffer* or *large buffer*); (b) were surveyed ≥6 years (i.e., ≥18% of the time) during the period 1984–2017 (Johnson et al., 2011); (c) contained at least two counts before and two counts after the year in which an individual fire event was recorded; and (d) did not have a 0 count (no males) in both survey years before the fire. These criteria were selected to ensure that leks were relevant to our study aims, had sufficient temporal replication for trend analysis, and had not become extirpated prior to a fire event.

To estimate prefire and postfire male lek attendance trends (i.e., whether the number of males at a lek is growing or declining over time), we implemented generalized linear models (GLMs) with a Poisson link function in the programming environment R (v. 3.4.1; R Foundation for Statistical Computing, Vienna, Austria). Male counts were used as the response variable while the year of the sage‐grouse count and the fire history (binary, where 0 = before fire and 1 = after fire) were used as explanatory variables. This allowed us to quantify annual male lek attendance before and after a fire event as:

(1) $$E\left(\ln Y\right)=\beta_{0}+\beta_{1}X_{1}+\beta_{2}X_{2}+\beta_{3}X_{1}X_{2}$$

where

*E*(ln *Y*) = expected natural logarithm of male sage‐grouse counts
*β*_0_ = intercept
*β*_1_, *β*_2_ and *β*_3_ = coefficients
*X*_1_ = year in which sage‐grouse count occurred (1984–2014)
$X_{2}=\left\{\begin{array}{c} 0\;\text{if}\;\text{before}\;\text{fire}\\ 1\;\text{if}\;\text{after}\;\text{fire} \end{array}\right.$

From this regression model, the prefire male attendance trend (slope of the regression line before fire) for each lek site can be estimated by simplifying Equation (1) with *X*_2_ = 0 to:

(2) $$E\left(\ln Y\right)=\beta_{0}+\beta_{1}X_{1}$$

To determine the postfire male attendance trend (slope of the regression line after fire) of each lek site, only counts after fire (*X*_2_ = 1) are included, simplifying Equation (1) to:

(3) $$E\left(\ln Y\right)=\left(\beta_{0}+\beta_{2}\right)+\left(\beta_{1}+\beta_{3}\right)X_{1}$$

Hence, these equations estimate the slopes of the regression line (male attendance trend) with *β*_1_ before fire (Equation 2) and with *β*_1_ + *β*_3_ after fire (Equation 3). To assess whether burned and unburned islands differ in the effects of fire on sage‐grouse male attendance trends, we aggregated the trend values (slopes) of all leks within each of the four fire categories (*unburned island*, *fire perimeter*, *small buffer* and *large buffer*) and compared the differences of the average population trends of each fire category before and after fire by applying *t* tests or Wilcoxon rank tests, depending on whether assumptions of normality were met.

### 2.5 Postfire habitat composition

To test to what extent postfire habitat composition affects sage‐grouse population trends after fire, we focused on three predictor variables (Table 1). In a preliminary analysis, we had initially explored seven predictor variables (vegetation type, vegetation cover, vegetation height, cheatgrass cover, elevation, precipitation, and temperature) that have previously been shown to be important to sage‐grouse ecology and population dynamics. However, after exploring multicollinearity among all covariates at all three spatial scales with Spearman's rank correlations (*r*) and the Variance Inflation Factor (VIF) of a full model, we subsequently only included covariates that were not highly correlated (*r* < 0.5) and had a VIF ≤ 3. This included three variables (Table 1): (a) average height of the remaining sagebrush vegetation (“vegetation height”) to characterize suitable habitat for sage‐grouse, (b) amount of unsuitable vegetation (“cheatgrass cover”), and (c) average elevation (“elevation”). Although the presence of invasive cheatgrass is often related to low elevation as it requires relatively dry and warm conditions (Chambers, Pyke, & Maestas, 2014), we could not detect a strong relationship between cheatgrass cover and elevation in our dataset (*r* of 0.16, 0.01, and −0.03 at 0.8 km, 6.4 km, and 18 km scales, respectively). This could reflect that cheatgrass cover in our study area is more strongly influenced by fire history than by regional climate (Jessop & Anderson, 2007). We also did not include unburned area as a predictor variable because it was strongly correlated with vegetation height (*r* of 0.76, 0.80 and 0.56 at 0.8 km, 6.4 km and 18 km scales, respectively), possibly because the burning of sagebrush strongly diminishes vegetation height (Baker, 2006). We used vegetation height rather than unburned area because it was a stronger predictor variable in univariate analyses at all three spatial scales.

**Table 1:** Habitat covariates used for explaining postfire sage‐grouse male attendance trends in relation to habitat features in southeastern Oregon, USA

Habitat variable	Rationale/hypotheses	Literature sources
Vegetation height	Sage‐grouse utilize higher stands of sagebrush vegetation because it offers more cover and food during winter More successful sage‐grouse nests are placed under tall sagebrush with high foliar cover	Connelly, Schroeder, et al. (2000) and Connelly, Reese, et al. (2000) Holloran et al. (2005)
Cheatgrass cover	Sage‐grouse avoid cheatgrass because it offers inadequate nesting cover Cheatgrass is often associated with high landscape disturbance (e.g., fire and loss of habitat)	Crawford et al. (2004) Johnson et al. (2011) Knick et al. (2013)
Elevation	Sage‐grouse often utilize higher elevation areas in summer because they have higher plant productivity, a longer growing season and higher forb and insect abundance than lower elevation areas Higher elevation areas are more resilient to disturbances such as fire	Chambers et al. (2014) Drut et al. (1994) Fischer, Reese, and Connelly (1996)

Note

Represented are the habitat covariates, together with hypothesized ecological importance (rationale) for sage‐grouse, and the literature sources which contain this information.

To quantify the postfire habitat variables, we utilized multitemporal vegetation maps as well as an elevation map from LANDFIRE (2017). The maps represent vegetation characteristics (i.e., vegetation type, height, and cover) and elevation above sea‐level (in meters). The LANDFIRE program categorizes vegetation height for each 30 m pixel in classes of 0.5 m (e.g., 0–0.5 m, 0.5–1 m, until >3 m). To quantify vegetation height, we used the mean value of each vegetation height class (e.g., 0.25 m for height class 0–0.5 m) and calculated the average vegetation height of sagebrush for all pixels around a lek that remained unburned after fire. For cheatgrass cover, we employed a 13‐year (2000–2013) cheatgrass database of the Great Basin which quantifies cheatgrass cover (0%–100%) at 250‐m resolution (Boyte, Wylie, & Major, 2016). We calculated the area surrounding leks with >8% cheatgrass cover because few active leks have >8% cheatgrass cover (Johnson et al., 2011). Elevation around a lek was determined by calculating the average elevation of all raster cells surrounding that lek. In all cases, the vegetation datasets used to determine postfire habitat composition were time specific and matched the period when a lek was first burned.

To test for spatial scale effects on habitat selection, we calculated all habitat variables with three distances (radii) around each lek: 0.8 km (2 km^2^), 6.4 km (129 km^2^), and 18 km (1,018 km^2^). The 0.8‐km distance was selected as the finest scale because it represents habitat characteristics that may impact breeding and nesting of sage‐grouse, which are often located in the direct vicinity of leks (Fremgen et al., 2017; Walker, Naugle, & Doherty, 2007). The 6.4‐km distance was used as an intermediate scale to reflect fire effects like loss of food sources and nesting habitat in the surroundings (Walker et al., 2007). This was reasonable because females generally distribute their nests within a radius of approximately 6.4 km around a lek (Hagen, 2011a). The 18‐km distance was chosen to reflect habitat composition at the landscape scale (Johnson et al., 2011). This scale is important because sage‐grouse often make seasonal movements at this scale (Connelly, Schroeder, Sands, & Braun, 2000).

We calculated postfire habitat composition (i.e., cheatgrass cover and vegetation height after fire), using the MTBS fire perimeter dataset to simulate the effect of burning on vegetation distribution. We delineated burned areas as all known fire perimeters in the year a specific lek was burned, as well as 17 years prior to the year in which a fire burned a lek. This was done because the earliest analyzed lek burned in 2001, which is 17 years after the earliest documented fire in the MTBS fire perimeter dataset in 1984. Furthermore, as effects of fire in sagebrush ecosystems are long term, with vegetation recovery times between 35 and 120 years (Baker, 2006), areas that have been burned for 17 years will most likely be still unsuitable for sage‐grouse nesting (Nelle, Reese, & Connelly, 2000). In the case of cheatgrass cover, burned areas around a lek with elevation <2,000 m were categorized as having unsuitable cheatgrass cover (>8% cover) for sage‐grouse because burned areas are highly susceptible to invasion by cheatgrass (Chambers, Roundy, Blank, Meyer, & Whittaker, 2007; Jessop & Anderson, 2007). Burned areas >2,000 m in elevation were categorized as having suitable cheatgrass cover (<8% cover), because cheatgrass does not seem to invade above this elevation (Boyte et al., 2016). Vegetation height of burned areas and unsuitable vegetation types, such as forests and juniper woodlands, was set to 0 m, as sagebrush stands often burn completely, leaving no remnant vegetation after fire (Baker, 2006).

We used ordinary least squares multiple linear regression models to assess how postfire habitat composition affects male attendance at leks after fire. We used the predicted postfire sage‐grouse male attendance trends (i.e., the estimated slopes after fire, see above Equation 3) as response variable and vegetation height, cheatgrass cover and elevation (all calculated with a radius of 0.8 km, 6.4 km, and 18 km, respectively) as predictor variables. All predictor variables were standardized to mean = 0 and *SD* = 1 to facilitate the interpretation and comparison of coefficients. We then performed a model selection in which all possible models nested within the global model (i.e., having all three predictor variables at all three scales) were fitted and ranked based on the Akaike Information Criterion for small sample sizes (AIC_c_) and Akaike weights *w_i_* (Burnham & Anderson, 2002). The parameters of all models were then weighted and averaged. Since we used *w_i_* to weigh the parameter estimates, models with very low support will only have a small influence on the averaged parameter estimates (Johnson & Omland, 2004). The model averaging allowed us to assess the relative importance of each habitat variable at each spatial scale in explaining postfire male attendance trends of sage‐grouse. We considered confidence intervals (CI) of average coefficients not including zero to indicate a strong statistically significant effect on postfire male attendance trends. All statistical analyses were performed in R, using the “MUMIn” package (Bartón, 2018).

## 3 RESULTS

### 3.1 Lek count data and fire history

In the study area, the ODFW has documented 756 leks that were surveyed at least once in the period 1984–2017. Of these leks, 33 were located inside of unburned islands, 106 inside fire perimeters, 133 leks in the small buffer, and 256 leks in the large buffer. After applying the four selection criteria for population trend analysis, a total of 39 leks remained (9 in unburned islands, 8 in fire perimeters, 5 in small buffers, and 17 in large buffers (Figure 3). During the period 1984–2014, a total of 17,300 km^2^ (23% of the study area) was burned. Within the 317 fire perimeters, an average of 54.6 km^2^ ± 163 km^2^ (10% of the area) consisted of unburned islands (in total *n* = 247,968), although the percentage of unburned islands within individual fire perimeters ranged widely from 2% to 78%, with a median unburned area of 8.3%. The amount of unburned area surrounding the 9 unburned island leks was 0.72 km^2^ ± 0.18 km^2^ (36% ± 9%) within a radius of 0.8 km around leks.

### 3.2 Population trend analysis

**Figure 4:**
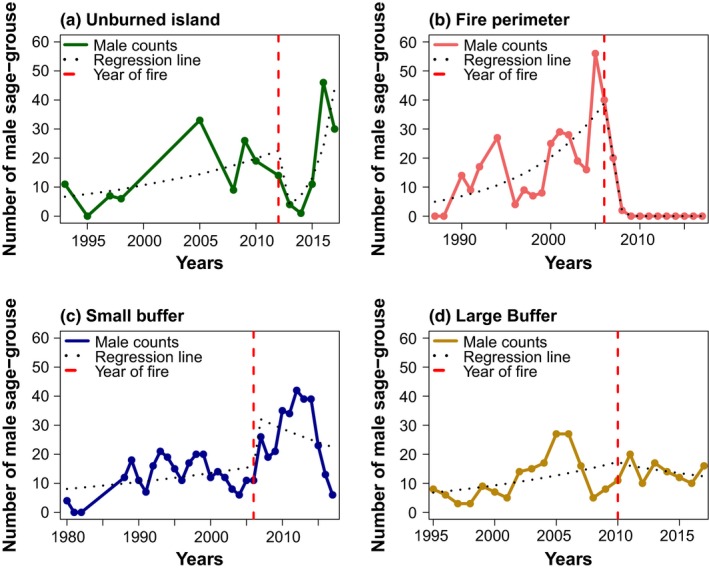
Examples of typical prefire and postfire male attendance trends of greater sage‐grouse (*Centrocercus urophasianus*) leks in four fire categories: (a) unburned island, (b) fire perimeter, (c) small buffer, and (d) large buffer. The graphs show (observed) male counts over time and (fitted) regression lines before and after a fire event. The sites are (a) Dry Creek #2, (b) Fields Creek, (c) Whiskey Springs #1, and (d) Lone Pine Road, representing an unburned island, fire perimeter, small buffer, and large buffer lek, respectively

**Figure 5:**
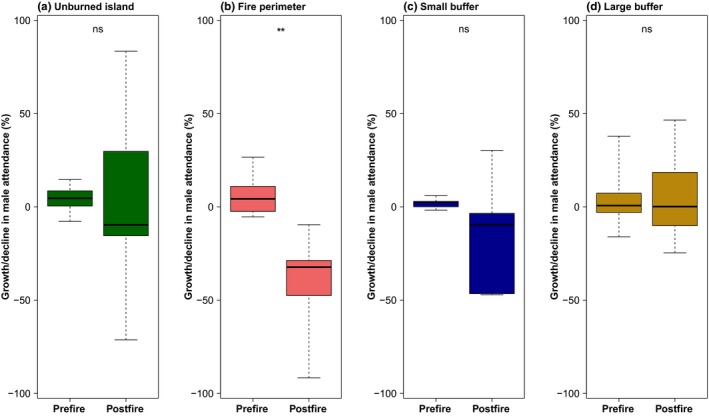
Boxplots summarizing prefire and postfire male attendance trends of greater sage‐grouse (*Centrocercus urophasianus*) across all leks in a specific fire category. (a) Unburned islands (*n* = 9; *t* = −0.11, *df* = 8.30, *p* = 0.91), (b) fire perimeter (*n* = 8; *t* = *4.84*, *df* = 9.52, *p* < 0.001), (c) small buffer (*n* = 5; *t* = 4.07, *df* = 9.52, *p* = 0.30), and (d) large buffer (*n* = 17; *W* = 152, *p* = 0.81). ** indicates a statistically significant difference (*p* < 0.001) before and after fire. “ns” means not statistically different. Statistical results represent mean difference tests (either *t* test or Wilcoxon rank test)

Using the GLMs of yearly peak male counts of sage‐grouse, we extracted pre‐ and postfire trends of male lek attendance (i.e., estimated slopes of the regression lines before and after fire) for each of the 39 leks (see examples in Figure 4). Within unburned islands (*n* = 9), male lek attendance trends did not show a statistically significant difference before and after fire (Figure 5a), suggesting that fire did not strongly decrease population sizes within unburned islands at leks included in our study. In several cases, postfire male lek attendance trends in unburned islands even showed a strong population increase after fire (Figure 4a), although one lek also showed a strong population decline. Within fire perimeters, sage‐grouse populations often crashed (Figure 4b) and male lek attendance trends were significantly lower after fire (Figure 5b), suggesting that fire has a strong negative effect on sage‐grouse population dynamics. Within the small buffer area (50 m–1.5 km away from fire perimeters), male lek attendance trends tended to decline after fire (Figure 4c), but the average postfire male lek attendance trend was not significantly different from the prefire trend (Figure 5c). In the large buffer area (control), populations were unaffected by fire with both prefire and postfire male lek attendance trends being relatively stable (Figure 4d) and showing no statistically significant changes after fire (Figure 5d).

### 3.3 Postfire habitat composition

**Figure 6:**
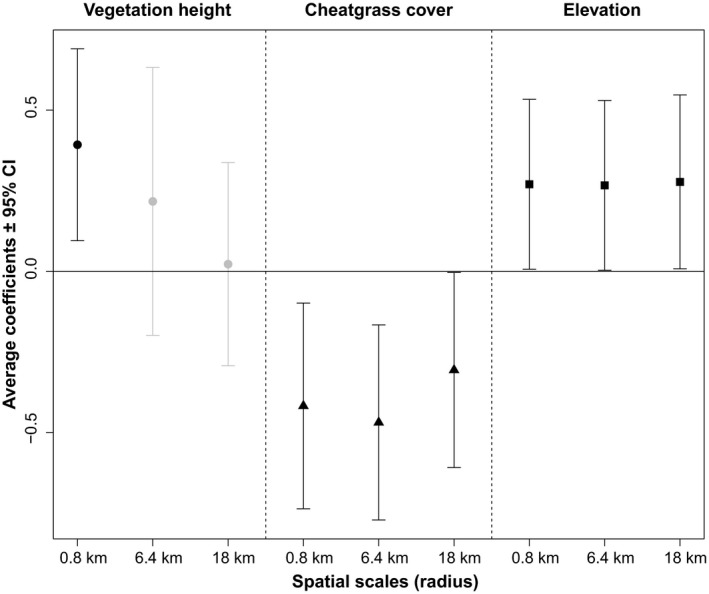
Average coefficients ± 95% confidence intervals (CI) of habitat variables (vegetation height, cheatgrass cover, and elevation) at three spatial scales (0.8 km, 6.4 km, and 18 km) showing their effect on postfire trends of male lek attendance (*β*_1_ + *β*_3_ as described in Equation 3). All possible models were ranked on the basis of Akaike weights (*w_i_*), and parameters of the full candidate set of models were then averaged. CI of parameters not overlapping 0 (shown in black) indicate a statistically significant effect on the postfire male attendance trend. CI overlapping 0 (shown in gray) indicates no statistically significant effect on the postfire male attendance trend

Out of the 39 leks with male lek attendance trend estimates, a total of 32 could be analyzed in relation to postfire habitat composition, as six leks (Long Dam, Maupin Spring #1, Water Trough, Hilltop #1, Blizzard #2 and Roy Reservoir) burned in a year for which no vegetation datasets were available (either prior to 2001 or after 2013), and one lek (Virtue #2) was located outside the extent of the cheatgrass cover maps. Using the three predictor variables (vegetation height, cheatgrass cover, and elevation), we fitted a total of 63 models to explain postfire sage‐grouse male attendance trend in relation to habitat features (Table S1). After model averaging, vegetation height had a strong positive effect on male postfire lek attendance trends at the finest spatial scale (0.8 km), but was not statistically significant at intermediate or landscape scales (Figure 6; Table 2). This suggested that (tall) sagebrush vegetation of unburned areas in the direct vicinity around leks has a positive influence on postfire population growth of sage‐grouse.

**Table 2:** Model‐averaged estimates of standardized regression coefficients (*β*) and standard errors (*SE*), 95% confidence intervals (CI) and *p*‐values for models explaining effects of habitat composition after fire on postfire population trends (i.e., regression slopes after fire) of sage‐grouse lek populations in southeastern Oregon, USA (between 2001 and 2017)

Variable	*β*	*SE*	Lower CI	Upper CI	*p*‐value
Vegetation height 0.8 km	0.3925	0.1460	0.0948	0.6901	0.0098
Vegetation height 6.4 km	0.2167	0.2053	−0.1987	0.6321	0.3066
Vegetation height 18 km	0.0221	0.1544	−0.2929	0.3371	0.8905
Cheatgrass cover 0.8 km	−0.4176	0.1567	−0.7366	−0.0985	0.0103
Cheatgrass cover 6.4 km	−0.4688	0.1486	−0.7714	−0.1662	0.0023
Cheatgrass cover 18 km	−0.3063	0.1481	−0.6087	−0.0038	0.0471
Elevation 0.8 km	0.2699	0.1289	0.0064	0.5333	0.0446
Elevation 6.4 km	0.2665	0.1289	0.0031	0.5296	0.0473
Elevation 18 km	0.2773	0.1319	0.0077	0.5469	0.0438

Note

All possible models were ranked using the Akaike Information Criterion for small sample sizes (AIC_c_), and parameters were then averaged for the full candidate set of models.

Cheatgrass cover showed a strong and statistically significant negative effect on postfire male attendance at all scales (Figure 6; Table 2), suggesting that the dominance of this invasive grass species may hamper the recovery and persistence of lek populations after fire. Elevation showed positive effects on postfire attendance trends at all three scales (Figure 6), indicating that lek populations at elevated sites show the strongest population growth after fire.

## 4 DISCUSSION

Our results show that male attendance of sage‐grouse at leks is negatively affected by fire when leks are located in burned areas inside fire perimeters. In contrast, leks in unburned islands or in areas far outside of fire perimeters showed predominantly stable or increasing population trends. Our results further demonstrate that vegetation height of unburned sagebrush habitat and elevation is positively associated with sage‐grouse population trends, whereas cover of cheatgrass shows a negative association. These findings support the hypothesis that unburned islands may serve as fire refugia for sage‐grouse, mitigating the negative effects of fire on lek attendance.

### 4.1 Population trends

Unburned islands may serve as refugia for wildlife populations because species can retreat to them during fire and repopulate burned areas after fire (Meddens, Kolden, Lutz, Smith, et al., 2018). Our results show that most leks located in unburned islands have stable or increasing attendance trends after fire. While our sample size is low, and a broad generalization may be too premature, we only found one lek in unburned islands (out of nine) that showed a strong decline in male attendance (see discussion below). The observed persistence of sage‐grouse populations within unburned islands may be attributed to several factors. First, unburned islands have higher nesting cover than burned areas, which can positively influence nesting success (Holloran et al., 2005). Second, important food sources for sage‐grouse such as forbs and insects are higher in unburned areas than in burned areas (Miller & Eddleman, 2001; Rickard, 1970). Third, recovery of vegetation, such as herbs and grasses, at the edge of unburned islands is often enhanced because seed dispersal from unburned islands allows for recolonization and provides enough forage and cover for sage‐grouse after fire (Foster et al., 2019; Longland & Bateman, 2002). These factors may enhance the successful breeding of sage‐grouse at leks in unburned islands. Although movement between leks is uncommon (e.g., Fremgen et al., 2017; Gibson, Blomberg, Atamian, & Sedinger, 2014), male sage‐grouse may experience lower lek fidelity in disturbed and fragmented landscapes as compared to intact habitats (Foster et al., 2019; Schroeder & Robb, 2003). As a result, the apparent high persistence within unburned islands may also be partly caused by postfire movements of male sage‐grouse from burned leks to unburned island leks. Telemetry data would be needed to confirm such an effect.

Unburned islands vary in their characteristics. For instance, most unburned islands in our dataset are relatively small (on average 0.01 ± 0.21 km^2^), but the additional number of unburned islands in the surrounding landscape of a lek may also be important. For instance, it is known that patch characteristics such as size, distribution, density, and shape may affect the functionality of unburned islands for survival of wildlife species (e.g., Chalfoun, Thompson, & Ratnaswamy, 2002; Longland & Bateman, 2002). The effect of these spatial characteristics of unburned islands for the persistence of sage‐grouse may warrant future research. Furthermore, home range sizes of sage‐grouse are up to 30 km^2^ (Connelly, Schroeder, et al., 2000), and sage‐grouse therefore cannot fulfil their entire life history within a single unburned island. Hence, habitat and landscape characteristics around leks will be relevant for population dynamics (see discussion below). Variation in environmental conditions during fires (e.g., amount of sagebrush cover, previously established cheatgrass, fuel moisture and wind speed/direction; Pyle & Crawford, 1996; Sapsis & Kauffmann, 1991) may influence the intensity of fires and consequently play an important role in determining the functionality of unburned islands as fire refugia for sage‐grouse (Baker, 2006; Meddens, Kolden, Lutz, Smith, et al., 2018). Four of the analyzed unburned island leks were situated within the same fire perimeter (Holloway Fire) which is known to be largely of moderate fire intensity (Foster, 2016). This may have positively affected the postfire habitat composition for the persistence of sage‐grouse as opposed to high intensity fires because moderate fire intensities might kill the whole plants. However, the survival of sagebrush may also be unrelated to fire intensity because sagebrush mortality can already occur at low fire intensities (Baker, 2006; Sapsis & Kauffmann, 1991).

While unburned islands can help to mitigate the negative effects of fire on sage‐grouse by enhancing nesting cover and food sources, carrying capacity in the landscape is still lower after fire (Foster, 2016). Subsequent fire frequency in burned areas may further increase due to the invasion of cheatgrass (Jessop & Anderson, 2007) and fragmentation of suitable sagebrush habitat may have further negative effects on population dynamics of sage‐grouse. Predation is a key factor determining sage‐grouse mortality in intact sagebrush ecosystems (Hagen, 2011b), and the increase in habitat edges through fire will result in habitat mosaics (Wiens, 1995) where predation can increase at the edges of unburned islands (Andren & Angelstam, 1988; Chalfoun et al., 2002; Šálek, Kreisinger, Sedláček, & Albrecht, 2010). Also, sage‐grouse fitness may be reduced due to limited diet and/or physiological stress after habitat loss (Hovick, Elmore, Wallred, Fuhlendorf, & Dahlgren, 2014). Hence, unburned islands could potentially also function as ecological traps (Gates & Gysel, 1978) because they force sage‐grouse to select lower‐quality habitats that have increased rates of predation and fitness costs (Battin, 2004). This effect may be exacerbated by the high site fidelity of sage‐grouse to leks (Fremgen et al., 2017), although some other research suggests that nest site fidelity is reduced when sage‐grouse inhabit disturbed and fragmented landscapes compared to intact habitats (e.g., Foster et al., 2019; Schroeder & Robb, 2003).

### 4.2 Postfire habitat composition

We found that suitable habitat (vegetation height of unburned sagebrush), unsuitable habitat (cheatgrass cover), and elevation can explain postfire male attendance trends at fine (0.8 km), intermediate (6.4 km) and landscape (18 km) spatial scales around leks. Besides cheatgrass cover at an intermediate scale, the strongest predictor was vegetation height of sagebrush at the fine spatial scale. This may be attributed to sage‐grouse preferentially nesting under sagebrush (Fremgen et al., 2017; Walker et al., 2007), where the most successful nests are placed under tall sagebrush with high foliar cover (Holloran et al., 2005). Because vegetation height was strongly correlated with unburned area at the local scale, this effect may also be attributed to the general presence of intact remnant sagebrush habitat in the direct vicinity around leks. The habitat preference of tall and intact sagebrush may also explain why male attendance at one of the unburned island leks decreased dramatically after fire, as at the finest scale, only 5% of the area surrounding this lek was composed of unburned sagebrush vegetation.

Our results also showed that postfire male attendance trends are negatively associated with cheatgrass cover and positively associated with elevation, from local to landscape scales. Sage‐grouse tend to avoid areas invaded by cheatgrass because they offer poor nesting cover (Crawford et al., 2004) and because they are associated with high disturbance and more frequent fires (Miller et al., 2011). This reduces habitat extent and habitat quality for the sage‐grouse (Connelly, Reese, et al., 2000; Connelly, Schroeder, et al., 2000). Invasion by cheatgrass is dependent on environmental factors such as temperature and precipitation, and higher elevation sagebrush ecosystems are less susceptible to cheatgrass establishment (Chambers et al., 2014). Moreover, areas higher in elevation show higher plant productivity, longer growing seasons, and higher forb and insect abundance compared to areas of lower elevation (Drut, Crawford, & Gregg, 1994). Finally, fires at higher elevation may burn patchier than those at low elevation, resulting in greater proportions of remnant intact habitat (Meddens, Kolden, Lutz, Abatzoglou, et al., 2018; Meddens, Kolden, Lutz, Smith, et al., 2018). This may benefit the recovery and persistence of sage‐grouse populations after fire. Our results reflect this effect, as four unburned island leks with the most stable postfire male attendance trends also occurred at the highest elevation of all analyzed leks.

In contrast to our results, increasing elevation (and thus, increasing precipitation and decreasing temperatures) does not necessarily always result in less cheatgrass cover or lower fire risk. For example, research by Britton and Clark (1985) found that a minimum of 20% sagebrush cover and approximately 300 kg/ha of herbaceous fuel might be required to set fire to sagebrush ecosystems. Accordingly, in low elevation areas, where warm and dry conditions are dominant, reduced fine‐fuel production may lower fire risk and lead to formation of unburned islands (Baker, 2006). Moreover, growth of cheatgrass is physiologically limited at lower elevations due to frequent low precipitation years (Chambers et al., 2014), and thus spreads most optimally at mid elevations under relatively moderate precipitation and temperature (Chambers et al., 2007). Resistance of higher elevation sagebrush ecosystems to wildfire and cheatgrass invasion may also reduce with future climate change, where higher temperatures, more irregular precipitation events, and longer and more frequent wildfire seasons may facilitate spread of cheatgrass into these areas (Bradley, 2009; Westerling, Hidalgo, Cayan, & Swetnam, 2006). As a result, the potential refugia effect of higher elevation sagebrush ecosystems that was observed during this study may change in the future.

Many other factors may also be important for the recovery and persistence of sage‐grouse after fire. For example, sage‐grouse lek density is negatively related to distance to mesic resources (Donnelly, Naugle, Hagen, & Maestas, 2016), as areas like upper elevation mesic sagebrush communities are important for sage‐grouse during brood‐rearing periods (Atamian, Sedinger, Heaton, & Blomberg, 2010). Furthermore, presence and density of juniper and the expansion of this species into sagebrush ecosystems can alter fire regimes and reduce habitat extent and habitat suitability for sage‐grouse (Baruch‐Mordo et al., 2013; Miller et al., 2011). The postfire recovery of native grasses, litter, and herbaceous cover may also play a role because sage‐grouse utilizes these for cover and foraging (Beck, Connelly, & Reese, 2009; Foster et al., 2019; Longland & Bateman, 2002). Finally, prefire and postfire restoration efforts in the vicinity of leks, such as fuel breaks, may also influence population persistence of the sage‐grouse (Murphy et al., 2013). We suggest that these factors require attention in future studies to better understand the determinants of postfire recovery and persistence of sage‐grouse populations.

### 4.3 Management implications for sage‐grouse

Our results suggest two key management implications for sage‐grouse. First, due to the strict requirements of sage‐grouse for intact sagebrush habitat, prefire efforts should be undertaken to inhibit the spread and size of wildfires within sage‐grouse habitat. Moreover, sagebrush has long recovery times after fire, and sagebrush habitat is highly prone to invasion by cheatgrass after fire (Chambers et al., 2007; Jessop & Anderson, 2007). Management actions could include control of invasive and encroaching species like cheatgrass and juniper into sage‐grouse habitat because they compete with sagebrush and negatively alter fire regimes by creating larger and more severe fires (Miller et al., 2011). Second, management actions should be carried out to preserve sage‐grouse habitat in the direct vicinity of known active leks. Judicious development of fuel breaks should be undertaken to decrease fire extents and thereby allow the protection of sage‐grouse habitat (Murphy et al., 2013). Fuel breaks could also be used to create unburned islands around known active sage‐grouse leks which may help to increase the persistence of lek populations. However, before such restoration efforts are undertaken, the role of fuel breaks for reducing fire risk and increasing habitat extent and quality should be more thoroughly investigated (Shinneman et al., 2019). Protecting mature sagebrush habitat and preventing the spread of invasive plant species should remain as highest priority.

## 5 CONCLUSIONS

Few studies have investigated the effect of fire and fire refugia on temporal dynamics of wildlife populations like the sage‐grouse (Coates et al., 2015; Foster et al., 2019; Robinson et al., 2013). The results of our study therefore constitute some of the first quantified evidence of the importance of unburned islands in the persistence and recovery of wildlife populations, using time series data in relation to specific fire events. Since our study has a small sample size and focuses only on one wildlife species of the sagebrush ecosystems of the Great Basin of North America, we urge subsequent studies with more comprehensive time series data as well as focused on other wildlife species in fire‐prone ecosystems and their population trends in response to the spatiotemporal dynamics of fire. Since fire refugia for wildlife species are likely to become increasingly important under the expected future changes in fire activity and climate change (Meddens, Kolden, Lutz, Abatzoglou, et al., 2018; Meddens, Kolden, Lutz, Smith, et al., 2018), we urge to increase knowledge on the functioning of fire refugia for the persistence of fire‐sensitive species. This will provide important information and aid decision‐making concerning land restoration and wildlife management in fire‐prone environments around the world.

## CONFLICT OF INTEREST

None declared.

## AUTHOR CONTRIBUTIONS

JS and AJHM conceived the ideas for this research and designed methodology together with AJM. LJF collected sage‐grouse lek data and AJHM developed the unburned island database. Data were analyzed by JS and interpreted together with WDK and LJF. The writing of the manuscript was led by WDK and JS. All authors contributed to the draft and gave final approval for publication.

## Data Availability

Data on the estimated attendance trends and extracted environmental covariates are available from the Dryad Digital Repository (https://doi.org/10.5061/dryad.30jh437). The unburned island database is available through https://www.sciencebase.gov/catalog/item/59a7452ce4b0fd9b77cf6ca0. Lek data can be requested through the ODFW.
