# Supplementary material for: The potential importance of unburned islands as refugia for the persistence of wildlife species in fire‐prone ecosystems
Source: Ecol Evol. 2019 Jul 4;9(15):8800–12. doi: 10.1002/ece3.5432 (PMC6686341; doi:10.1002/ece3.5432)
Supplement: Supplementary file 1 [file ECE3-9-8800-s001.docx]

# APPENDIX 1

**Table S1:** Overview of all fitted models to explain post-fire sage-grouse male attendance trend in relation to habitat features in south-eastern Oregon (2001–2014). Presented are the model number (No.), model input variables, the log-likelihood (LL), number of input variables (*K*), sample size (*n*), Akaike’s Information Criterion for small sample sizes (AIC_c_), delta-AIC (ΔAIC) and Akaike weights (*w_i_*). Models are represented in order of increasing ΔAIC units, starting with the best approximating model (with AICc = 308.02).

| No. | Model input variables | *LL* | *K* | *n* | AIC_c_ | ΔAIC | *w_i_* |
| --- | --- | --- | --- | --- | --- | --- | --- |
| 1 | Elevation 0.8 km + Vegetation height 0.8 km + Cheatgrass cover 6.4 km | -147.86 | 3 | 32 | 308.02 | 0.00 | 0.20 |
| 2 | Elevation 6.4 km + Vegetation height 0.8 km + Cheatgrass cover 6.4 km | -147.92 | 3 | 32 | 308.15 | 0.12 | 0.19 |
| 3 | Elevation 18 km + Vegetation height 0.8 km + Cheatgrass cover 6.4 km | -147.94 | 3 | 32 | 308.18 | 0.16 | 0.19 |
| 4 | Vegetation height 0.8 km + Cheatgrass cover 6.4 km | -150.29 | 2 | 32 | 310.07 | 2.04 | 0.07 |
| 5 | Elevation 18 km + Vegetation height 0.8 km + Cheatgrass cover 0.8 km | -149.22 | 3 | 32 | 310.75 | 2.72 | 0.05 |
| 6 | Elevation 0.8 km + Vegetation height 0.8 km + Cheatgrass cover 0.8 km | -149.23 | 3 | 32 | 310.76 | 2.74 | 0.05 |
| 7 | Elevation 6.4 km + Vegetation height 0.8 km + Cheatgrass cover 0.8 km | -149.46 | 3 | 32 | 311.22 | 3.20 | 0.04 |
| 8 | Elevation 18 km + Cheatgrass cover 6.4 km | -151.59 | 2 | 32 | 312.65 | 4.63 | 0.02 |
| 9 | Elevation 6.4 km + Cheatgrass cover 6.4 km | -151.75 | 2 | 32 | 312.98 | 4.96 | 0.02 |
| 10 | Vegetation height 0.8 km + Cheatgrass cover 18 km | -151.85 | 2 | 32 | 313.19 | 5.16 | 0.02 |
| 11 | Elevation 0.8 km + Cheatgrass cover 6.4 km | -152.02 | 2 | 32 | 313.53 | 5.50 | 0.01 |
| 12 | Vegetation height 0.8 km + Cheatgrass cover 0.8 km | -152.40 | 2 | 32 | 314.29 | 6.26 | 0.01 |
| 13 | Elevation 18 km + Vegetation height 0.8 km + Cheatgrass cover 18 km | -151.01 | 3 | 32 | 314.34 | 6.31 | 0.01 |
| 14 | Cheatgrass cover 6.4 km | -153.88 | 1 | 32 | 314.62 | 6.59 | 0.01 |
| 15 | Elevation 18 km + Vegetation height 0.8 km | -152.61 | 2 | 32 | 314.70 | 6.67 | 0.01 |
| 16 | Elevation 18 km + Vegetation height 0.8 km + Cheatgrass cover 0.8 km | -151.29 | 3 | 32 | 314.88 | 6.86 | 0.01 |
| 17 | Elevation 6.4 km + Vegetation height 0.8 km + Cheatgrass cover 18 km | -151.29 | 3 | 32 | 314.89 | 6.86 | 0.01 |
| 18 | Elevation 18 km + Cheatgrass cover 0.8 km | -152.73 | 2 | 32 | 314.93 | 6.91 | 0.01 |
| 19 | Elevation 6.4 km + Vegetation height 6.4 km + Cheatgrass cover 0.8 km | -151.36 | 3 | 32 | 315.04 | 7.01 | 0.01 |
| 20 | Elevation 0.8 km + Vegetation height 6.4 km + Cheatgrass cover 0.8 km | -151.38 | 3 | 32 | 315.08 | 7.05 | 0.01 |
| 21 | Elevation 18 km + Vegetation height 6.4 km + Cheatgrass cover 0.8 km | -151.39 | 3 | 32 | 315.09 | 7.07 | 0.01 |
| 22 | Elevation 18 km + Vegetation height 6.4 km + Cheatgrass cover 6.4 km | -151.45 | 3 | 32 | 315.20 | 7.18 | 0.01 |
| 23 | Elevation 6.4 km + Vegetation height 6.4 km + Cheatgrass cover 6.4 km | -151.52 | 3 | 32 | 315.36 | 7.33 | 0.01 |
| 24 | Elevation 18 km + Vegetation height 18 km + Cheatgrass cover 6.4 km | -151.58 | 3 | 32 | 315.47 | 7.44 | 0.00 |
| 25 | Elevation 6.4 km + Vegetation height 18 km + Cheatgrass cover 6.4 km | -151.75 | 3 | 32 | 315.81 | 7.78 | 0.00 |
| 26 | Elevation 6.4 km + Cheatgrass cover 0.8 km | -153.17 | 2 | 32 | 315.82 | 7.80 | 0.00 |
| 27 | Elevation 0.8 km + Vegetation height 6.4 km + Cheatgrass cover 6.4 km | -151.77 | 3 | 32 | 315.85 | 7.83 | 0.00 |
| 28 | Elevation 0.8 km + Cheatgrass cover 0.8 km | -153.29 | 2 | 32 | 316.06 | 8.03 | 0.00 |
| 29 | Elevation 6.4 km + Vegetation height 0.8 km | -153.43 | 2 | 32 | 316.34 | 8.31 | 0.00 |
| 30 | Elevation 0.8 km + Vegetation height 18 km + Cheatgrass cover 6.4 km | -152.02 | 3 | 32 | 316.35 | 8.33 | 0.00 |
| 31 | Elevation 0.8 km + Vegetation height 0.8 km | -153.48 | 2 | 32 | 316.44 | 8.42 | 0.00 |
| 32 | Vegetation height 6.4 km + Cheatgrass cover 6.4 km | -153.79 | 2 | 32 | 317.07 | 9.04 | 0.00 |
| 33 | Vegetation height 18 km + Cheatgrass cover 6.4 km | -153.84 | 2 | 32 | 317.16 | 9.14 | 0.00 |
| 34 | Elevation 18 km + Vegetation height 18 km + Cheatgrass cover 0.8 km | -152.43 | 3 | 32 | 317.17 | 9.15 | 0.00 |
| 35 | Vegetation height 0.8 km | -155.16 | 1 | 32 | 317.18 | 9.16 | 0.00 |
| 36 | Elevation 6.4 km + Vegetation height 18 km + Cheatgrass cover 0.8 km | -152.96 | 3 | 32 | 318.22 | 10.19 | 0.00 |
| 37 | Elevation 0.8 km + Vegetation height 18 km + Cheatgrass cover 0.8 km | -153.07 | 3 | 32 | 318.44 | 10.41 | 0.00 |
| 38 | Vegetation height 6.4 km + Cheatgrass cover 0.8 km | -154.72 | 2 | 32 | 318.92 | 10.89 | 0.00 |
| 39 | Cheatgrass cover 0.8 km | -156.16 | 1 | 32 | 319.18 | 11.16 | 0.00 |
| 40 | Elevation 18 km + Vegetation height 6.4 km | -155.05 | 2 | 32 | 319.58 | 11.55 | 0.00 |
| 41 | Vegetation height 6.4 km + Cheatgrass cover 18 km | -155.34 | 2 | 32 | 320.15 | 12.13 | 0.00 |
| 42 | Elevation 6.4 km + Vegetation height 6.4 km | -155.52 | 2 | 32 | 320.51 | 12.49 | 0.00 |
| 43 | Elevation 18 km + Vegetation height 6.4 km + Cheatgrass cover 18 km | -154.14 | 3 | 32 | 320.59 | 12.56 | 0.00 |
| 44 | Elevation 6.4 km + Vegetation height 6.4 km + Cheatgrass cover 18 km | -154.28 | 3 | 32 | 320.88 | 12.85 | 0.00 |
| 45 | Elevation 0.8 km + Vegetation height 6.4 km | -155.82 | 2 | 32 | 321.12 | 13.09 | 0.00 |
| 46 | Elevation 0.8 km + Vegetation height 6.4 km + Cheatgrass cover 18 km | -154.48 | 3 | 32 | 321.27 | 13.25 | 0.00 |
| 47 | Vegetation height 18 km + Cheatgrass cover 0.8 km | -156.10 | 2 | 32 | 321.68 | 13.65 | 0.00 |
| 48 | Vegetation height 6.4 km | -157.78 | 1 | 32 | 322.41 | 14.39 | 0.00 |
| 49 | Elevation 18 km | -159.43 | 1 | 32 | 325.71 | 17.68 | 0.00 |
| 50 | Cheatgrass cover 18 km | -159.70 | 1 | 32 | 326.25 | 18.23 | 0.00 |
| 51 | Elevation 18 km + Vegetation height 18 km | -158.47 | 2 | 32 | 326.42 | 18.39 | 0.00 |
| 52 | Elevation 18 km + Cheatgrass cover 18 km | -158.52 | 2 | 32 | 326.51 | 18.49 | 0.00 |
| 53 | Elevation 18 km + Vegetation height 18 km + Cheatgrass cover 18 km | -157.36 | 3 | 32 | 327.03 | 19.01 | 0.00 |
| 54 | Vegetation height 18 km + Cheatgrass cover 18 km | -158.79 | 2 | 32 | 327.05 | 19.03 | 0.00 |
| 55 | Elevation 6.4 km + Cheatgrass cover 18 km | -159.23 | 2 | 32 | 327.95 | 19.93 | 0.00 |
| 56 | Elevation 6.4 km | -160.77 | 1 | 32 | 328.39 | 20.37 | 0.00 |
| 57 | Elevation 0.8 + Cheatgrass cover 18 km | -159.46 | 2 | 32 | 328.41 | 20.39 | 0.00 |
| 58 | Elevation 6.4 km + Vegetation height 18 km + Cheatgrass cover 18 km | -158.17 | 3 | 32 | 328.66 | 20.63 | 0.00 |
| 59 | Elevation 0.8 km + Vegetation height 18 km + Cheatgrass cover 18 km | -158.42 | 3 | 32 | 329.15 | 21.13 | 0.00 |
| 60 | Elevation 0.8 km | -161.20 | 1 | 32 | 329.26 | 21.24 | 0.00 |
| 61 | Elevation 6.4 km + Vegetation height 18 km | -159.97 | 2 | 32 | 329.43 | 21.40 | 0.00 |
| 62 | Vegetation height 18 km | -161.67 | 1 | 32 | 330.20 | 22.17 | 0.00 |
| 63 | Elevation 0.8 km + Vegetation height 18 km | -160.43 | 2 | 32 | 330.33 | 22.31 | 0.00 |
